# Rapid Thermal Annealing of Double Perovskite Thin Films Formed by Polymer Assisted Deposition

**DOI:** 10.3390/ma13214966

**Published:** 2020-11-04

**Authors:** Hailin Wang, Carlos Frontera, Benjamín Martínez, Narcís Mestres

**Affiliations:** Institut de Ciència de Materials de Barcelona, ICMAB, Consejo Superior de Investigaciones Científicas, CSIC, Campus UAB, 08193 Bellaterra, Barcelona, Spain; hwang@icmab.es (H.W.); frontera@icmab.es (C.F.)

**Keywords:** functional oxides, double perovskites, B-site ordering, ferromagnetism, chemical growth methods, polymer-assisted deposition

## Abstract

The annealing process is an important step common to epitaxial films prepared by chemical solution deposition methods. It is so because the final microstructure of the films can be severely affected by the precise features of the thermal processing. In this work we analyze the structural and magnetic properties of double perovskite La_2_CoMnO_6_ and La_2_NiMnO_6_ epitaxial thin films prepared by polymer-assisted deposition (PAD) and crystallized by rapid thermal annealing (RTA). It is found that samples prepared by RTA have similar values of saturation magnetization and Curie temperature to their counterparts prepared by using conventional thermal annealing (CTA) processes, thus indicating low influence of the heating rates on the B-B’ site cationic ordering of the A_2_BB’O_6_ double perovskite structure. However, a deeper analysis of the magnetic behavior suggested some differences in the actual microstructure of the films.

## 1. Introduction

Most of the new technological applications of materials need to utilize thin films and heterostructures to take advantage of the varied benefits of miniaturization. Thus, preparation and characterization of thin films and heterostructures has become a very active area of research in which traditional high-vacuum deposition methods rival with more affordable chemical deposition methods. While traditional high-vacuum techniques, such as molecular beam epitaxy [1,2], radiofrequency sputtering [3,4], and pulsed laser deposition [5,6], offer clear benefits for growing thin films of metal oxides with high crystalline quality and excellent control over thickness and composition at the atomic-scale, chemical solution deposition (CSD) methods represent a more affordable alternative for extensive production of high quality functional oxide thin films [7,8,9]. The main benefits of this CSD methodology are the cheap and facile scalability of the deposition process. Additionally, CSD methods allow an easy control of the desired stoichiometry and film thickness with in situ doping possibilities, via the addition of a dopant to the precursor solution. Polymer-assisted deposition (PAD) is one of the CSD methods with major projection. PAD is based on very stable and homogeneous metal-polymer complex aqueous solutions. Towards this end, a water soluble polymer is employed to bind and stabilize the metal cations in the precursor solutions on the one hand and also to regulate the solution viscosity determining the film coating [10].

An important step, common to all CSD film growth methods, is the annealing process since the microstructure of the films can be severely affected by the precise features of the thermal processing [11,12,13]. In particular, the annealing ramp rate is significant for materials with a wide range of nucleation energies. In contrast to more usual low ramp rate (2–10 °C/min) annealing methods, in rapid thermal annealing (RTA) with heating rates 10–50 °C/s, the physical processes leading to densification and crystallization of the film are delayed to higher temperatures. On the contrary, when using slow heating ramps nucleation and growth start at lower temperatures and progresses as the temperature increases. This broad processing window often results in a wide nucleation range and distinct grain growth rates, which determine the final film microstructure and may lead to incoherently textured films. Conversely, if the film is rapidly heated, simultaneous nucleation occurs at high temperatures in a situation similar to that of isothermal annealing, which leads to a more uniform texture and grain size distribution [14].

Additionally, it is also expected that the RTA process will minimize possible diffusion reactions between films and substrates while enhancing the film densification and preventing the stabilization of intermediate phases, as already observed in the microelectronics industry where RTA is conventionally used [15]. According to these features, RTA might also be of interest to tune the final physical properties of complex oxide thin films, in particular it could be very interesting in the case of double perovskite oxides in which B-site cationic ordering plays a crucial role in their final physical properties. Double perovskite structures of the type A_2_BB’O_6_ are made of stacking single perovskite units and the unit cell is twice that of the perovskite. In the ideal double perovskite structure, in which A is a rare-earth metal or an alkaline earth metal cation and B and B’ are transition metal cations, a 3-D network of alternating BO_6_ and B’O_6_ octahedra exists. These materials have attracted considerable attention because of their particular ferromagnetic, electrical, and elastic properties [16]. Attaining a complete cationic ordering is demanding since the alternative occupancy of the B-B′-site cations can be influenced by several factors, such as processing conditions and ionic characteristics. It has been established that the larger the difference in size and charge of the B-B′ site cations, the more easy it is to grow a material with a higher degree of ordering [16]. Correspondingly, the final physical properties are strongly influenced by the degree of B-B’ site cationic ordering [17].

As previously shown [18,19], the PAD technique has been successfully used for the growth of high-quality double perovskite oxide thin films (La_2_CoMnO_6_ (LCMO) and La_2_NiMnO_6_ (LNMO)) with magnetic properties close to the optimal ones. It is well known that the magnetic properties of these compounds are extremely sensitive to B-B’site cationic ordering of Mn^4+^ and Co^2+^/Ni^2+^ ions.

In this work we have investigated the effect of fast heating rates on the magnetic properties of manganese-based double perovskite La_2_CoMnO_6_ and La_2_NiMnO_6_ thin films synthesized by using the PAD method. Results obtained from RTA samples are compared with those obtained in samples prepared by using conventional thermal annealing (CTA) processes. It is found that samples prepared by RTA have similar values of saturation magnetization and Curie temperature to those prepared by using CTA, thus indicating the low influence of the heating rates on the B-B’ site cationic ordering of the A_2_BB’O_6_ double perovskite structure. However, a deeper analysis of the magnetic properties suggests a slightly different microstructure. In particular, it is observed that antiphase boundaries in LCMO samples prepared by RTA can be easily suppressed by a subsequent annealing in an oxygen rich atmosphere, in contrast to what is observed in the counterpart samples processed by conventional annealing methods. In parallel with this, different behavior after zero field cooling-field cooling processes in *M*(*T*) curves is also observed, mainly in LNMO, thus also pointing to a different microstructure from that of samples prepared by using a conventional annealing process. 

## 2. Materials and Methods 

### 2.1. Precursor Solutions Preparation

The precursor solutions for the LCMO and LNMO thin films growth were produced by mixing previously synthesized solutions of La, Co, Mn and La, Ni, Mn respectively, bound to polymers using water as the solvent. First, individual solutions were prepared by using lanthanum(III) nitrate, cobalt(II) nitrate, nickel(II) chloride, and Mn(II) nitrate metal salts of high-purity (>99.9%). Branched polyethylenimine PEI, from Sigma Aldrich, Steinheim, Germany (average Mw ~25,000) was used as the binding polymer; and Ethylenediaminetetraacetic acid (EDTA), also provided by Sigma Aldrich, (Steinheim, Germany) was the complexing agent used. The different metal ion solutions were prepared by dissolving the proper metal salts in Milli-Q water and EDTA in a 1:1 molar ratio. The amount of PEI added to the solution was in a 1:1 mass ratio with EDTA. Each separate solution was filtrated using an Amicon^®^ filtration unit (Merck, Darmstadt, Germany) and 10 kDa filters, to remove non-coordinated cations and polymer fractions, and to obtain a homogeneous precursor solution. The retained fractions were analyzed by inductively coupled plasma (ICP) using Optima 4300™ DV ICP-OES Perkin-Elmer (Waltham, MA, USA) equipment, to precisely determine the cation concentration in the primary solutions. 

The obtained concentrations of the used solutions were [La] = 230.4 mM, [Co] = 146.4 mM, [Ni] = 272.6 mM, and [Mn] = 176.4 mM. From these primary solutions, the final precursor solutions with the desired La:Co:Mn 2:1:1, and La:Ni:Mn 2:1:1 stoichiometries were prepared and concentrated. The final cation concentration was adjusted to be 60–65 mM with respect to Mn. These conditions were chosen to be able to produce films in the 15–25 nm thickness range. Typical viscosity values were η ≈ 3–4 mPa s (measured with a DMA 4100 M Anton Paar (Ashland, VA, USA) densimeter, with a micro-viscometer module Lovis 2000 ME).

### 2.2. Thin Films Growth

The prepared precursor solutions were spin-coated on top of 0.5 × 0.5 cm^2^ (001)-SrTiO_3_ (STO) or (001)-LaAlO_3_ (LAO) substrates from Crystec, GmbH (Berlin, Germany). Previously, to create TiO_2_-terminated substrates with atomically flat terraces the as-received STO substrates were chemically etched and thermally treated [20]. Similarly, to create AlO_2_-terminated substrates with atomically flat terraces, the LAO substrates were thermally treated at high temperatures (950 °C) under oxygen flow.

The last step for the film growth is the thermal treatment of the spin-coated films under oxygen flow. This thermal annealing leads to elimination of the organic components at low temperatures, and to phase formation and crystallization at high temperatures. Rapid thermal annealing was achieved using an AS-Micro RTA furnace from Annealsys (Montpellier, France) under a controlled and stagnant oxygen atmosphere (loaded at 5 L/min for 2 min), and at temperatures from 800 to 1000 °C with dwell times in the 10 to 30 min range, and heating rates from 0.5 to 20 °C/s. Conventional thermal annealing was accomplished by using a tube furnace and heating ramps of several degrees Celsius per minute under oxygen flow to elude the formation of oxygen vacancies (oxygen flow rates between 100 and 600 mL/min).

### 2.3. Characterization of Structural and Physical Properties

The structural properties of the grown films were studied by X-ray diffraction and reflectivity employing a Bruker D8-Discover (Billerica, MA, USA) and a D5000-Siemens (Madison, WI, USA) diffractometers, with Cu-K_α1_ monochromatic radiation (1.5406 Å). The surface morphology of the epitaxial films was analyzed by atomic force microscopy (AFM) performed in tapping mode using an Asylum Research MFP-3D (Wiesbaden, Germany) microscope. DC magnetization measurements as a function of temperature and magnetic fields were performed using a superconducting quantum interference device (SQUID) from Quantum Design (San Diego, CA, USA). External magnetic fields were applied either parallel (in-plane (IP) configuration) or perpendicular (out-of-plane (OP) configuration) to the film/sample plane. The diamagnetic contribution of the substrate and other instrumental contributions were properly corrected [21]. The possible relative error when determining the saturation magnetization (*Ms*) was estimated to be approximately 5–8% and is predominantly ascribed to the error in the determination of the volume of the films. Once the thin film is grown, in most cases it is necessary to remove the accumulated material in the corners by chemical etching, to quantify with greater precision the values of the magnetic properties, and this introduces a non-negligible source of error. These patterns occur outside the circumference of the inscribed circle when spin coating on a square substrate, where radial uniformity vanishes [22].

## 3. Results and Discussion

### 3.1. Structural Characterization

**Figure 1:**
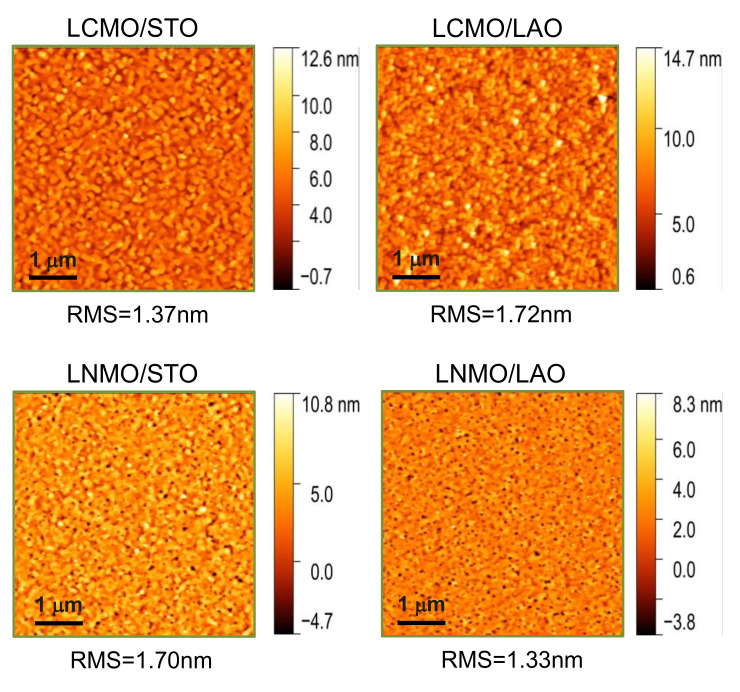
Atomic force microscopy surface topography images (5 × 5 μm^2^) of representative La_2_CoMnO_6_ and La_2_NiMnO_6_ thin films, with thickness values of about 20 nm, on top of SrTiO_3_ (STO) and LaAlO_3_ (LAO) substrates. Films thermally treated in rapid thermal annealing (RTA) conditions, 20 °C/s heating ramp, dwell time 20 min at 900 °C in static oxygen. In all cases, root mean square (RMS) roughness values are below 2 nm.

The surface morphology of the films was studied by atomic force microscopy (AFM). Figure 1 shows topography images of ~20 nm thick LCMO and LNMO thin films grown by RTA on top of STO and LAO substrates. All the films present flat surfaces with low values of RMS (below 2 nm), as indicated in the figure. This fact confirms that the PAD method using RTA thermal treatment is able to produce films with surface roughness similar to the ones obtained by pulsed laser deposition (PLD) or sputtering deposition methods.

**Figure 2:**
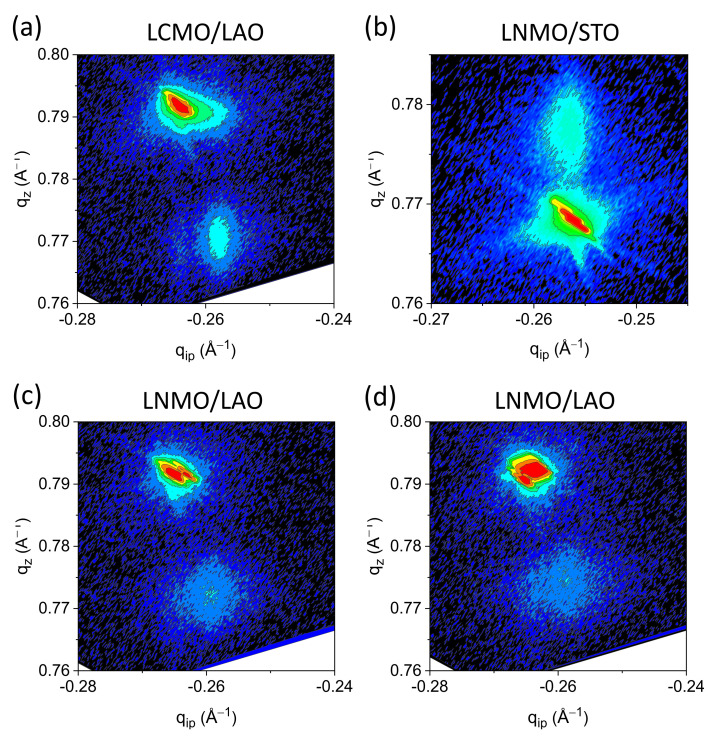
Reciprocal space maps around the (103) substrate reflections for (**a**) LCMO film grown on LAO; (**b**) LNMO film grown on STO; (**c**) LNMO film grown on LAO; treated in RTA conditions, 20 °C/s heating ramp, dwell time 20 min at 900 °C in static oxygen (**d**) LNMO film grown on LAO in RTA conditions, and subsequent high temperature conventional annealing in oxygen flux (5 °C/min, 750 °C, 120 min, 0.5 L/min oxygen).

The in-plane strain degree of the grown epitaxial films has been investigated by means of reciprocal space maps adjacent to the (103) substrate signal. Reciprocal space maps of LCMO films grown on STO substrate (not shown) reveal that these films grew fully strained without any measurable difference of the in-plane lattice parameters of film and substrate. Conversely, LCMO films grown on LAO were fully relaxed (see Figure 2a), and the in-plane lattice parameter extracted from the reciprocal space map is about *a* = 3.88 Å.

A parallel analysis was performed on LNMO films. Reciprocal space map examination shows that LNMO films grown on STO substrates are fully strained (Figure 2b). On the contrary, when the LNMO films are grown on LAO, the peak position of (103) reflection is clearly shifted from that of the LAO substrate along the q-_100_ axis (see Figure 2c). This fact is a direct consequence of the large lattice mismatch between the LNMO films and the LAO substrate (−2.31%), that causes a relaxation of the films structure. Moreover, the larger broadening observed in the LNMO film peak indicates that part of the film is fully relaxed and that some part is not. As can be observed in Figure 2d, this strained state is maintained even after a subsequent anneal at a high temperature under oxygen flow.

The measured strain behavior is similar to the one observed in PAD grown films using conventional thermal annealing [19] (see Supplementary Information Figure S1, for θ-2θ XRD spectra of representative LCMO/LAO and LNMO/LAO epitaxial thin films). Namely, epitaxial La_2_CoMnO_6_ and La_2_NiMnO_6_ thin films grow coherently and tensile strained on STO substrate owing to the fact that the lattice parameters of pseudo-cubic bulk La_2_CoMnO_6_ (*a_pc_* = 3.89 Å) [23] and La_2_NiMnO_6_ (*a_pc_* = 3.879 Å) [24], are close to the lattice parameter of STO (*a* = 3.905 Å). On the contrary, due to the large nominal lattice mismatch of the LCMO/LAO (−2.61%) and LNMO/LAO (−2.31%) arrangements, for film thickness values close to 20 nm, LCMO grows fully relaxed on LAO substrates (*a_pc_* = 3.791 Å), and LNMO grows partially relaxed on the same substrate. 

Moreover, rocking curves measured on the (002) reflections of the films have full width at half maximum (FWHM) values in the range of 0.12–0.17 degrees (see Supplementary Information Figure S2 for representative LNMO/STO and LNMO/LAO films). These relatively small FWHM values indicate that the RTA-grown films have a good crystallinity with some mosaicity.

### 3.2. Magnetic Properties

Ferromagnetic (FM) ordering in LCMO and LNMO double perovskites is generated by super-exchange interactions between Mn^4+^ and Co^2+^ (Ni^2+^) ions according to the Goodenough–Kanamori rules [25,26,27]. The corresponding spin-only theoretical values of saturation magnetization, *Ms*, of LCMO (Co^2+^ (3d^7^, t_2g_^5^ e^2^_g_; S = 3/2) and Mn^4+^ (3d^3^, t_2g_^3^e^0^_g_; S = 3/2)) and LNMO (Ni^2+^ (3d^8^, t_2g_^6^ e_g_^2^; S = 1) and Mn^4+^ (3d^3^, t_2g_^3^ e_g_^0^; S = 3/2)) are 6 and 5 μ_B_/f.u. respectively. However, if there is some disorder in the B-B’-site occupancy among Mn^4+^ and Co^2+^ (Ni^2+^) ions generating anti-site defects (ASD), i.e., a portion of Co (Ni) and Mn ions have their crystallographic sites interchanged, Co^2+^-O-Co^2+^ (Ni^2+^-O-Ni^2+^) and Mn^4+^-O-Mn^4+^ antiferromagnetic (AFM) interactions are introduced lowering the saturation magnetization value by a factor (1-2·X(ASD)), where X(ASD) is the fraction of ASD disorder [28]. Therefore, magnetic measurements are a very sensitive sensor to determine the amount of B-B’ site cationic ordering. The ordered occupancy of the B sublattice by Mn and Co (Ni) ions is difficult to achieve because it is affected by different factors, such as ionic characteristics and synthesis circumstances. In general, it is observed that the larger the difference in charge and ionic radii of the B-site cations, the higher the grade of ordering achieved. According to this, we will proceed to analyze both the temperature dependence, *M*(*T*), and the magnetic field dependence, *M*(*H*), of magnetization in samples prepared by RTA in comparison with similar samples prepared by traditional thermal processing. 

Table 1 summarizes the different RTA growth conditions and the subsequent CTA treatments for the samples investigated, together with the extracted values of Curie temperature *Tc* and saturation magnetization *Ms*. 

**Figure 3:**
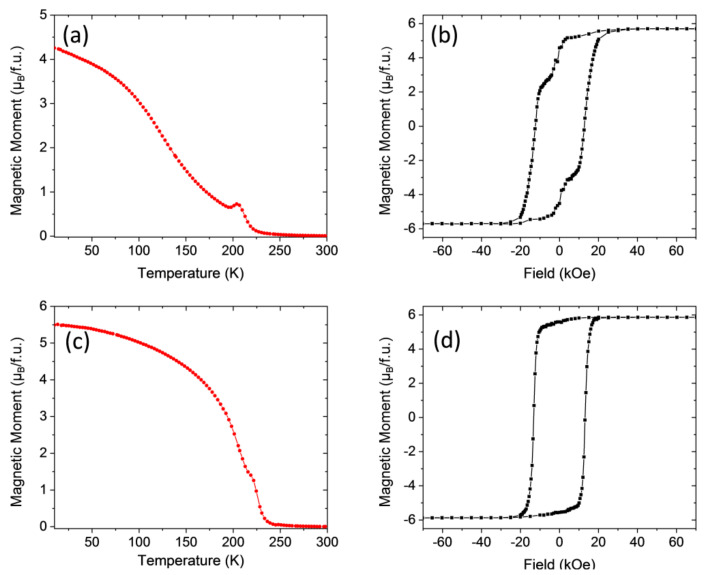
(**a**) Magnetization vs. temperature of LCMO/STO–1 film measured after field cooling with a magnetic field of 1 kOe applied parallel to (001)-STO (out of plane). (**b**) M–H loop of the same sample recorded at 10 K, magnetic field H applied out of plane. (**c**) Temperature dependence of the magnetization under an applied magnetic field of 1 kOe (out of plane), of sample LCMO/STO–1R. (**d**) M–H loop of sample LCMO/STO–1R recorded at 10 K, magnetic field H applied out of plane.

We will analyze first the results obtained in the LCMO system. In Figure 3 *M*(*T*) and *M*(*H*) curves corresponding to the LCMO/STO–1 sample processed in static oxygen atmosphere with a heating ramp of 20 °C/s and a dwell time of 20 min at 900 °C are reported. The field cooled temperature dependence of the magnetization *M*(*T*) under an external magnetic field of 1 kOe applied along the easy magnetization direction, that for the LCMO samples is perpendicular to the film plane, i.e., out-of-plane (OP) configuration, is shown in Figure 3a. The *M*(*T*) curve exhibits a non-monotonic behavior with a local minimum around *T* ≈ 200 K and a paramagnetic to ferromagnetic transition temperature *Tc* ≈ 230 K, which often has been interpreted as a proof of the existence of two different phases in the sample [29,30,31]. Nevertheless, it is worth mentioning here that the existence of two different magnetic phases will require different oxidation states of Mn and Co from that of Mn^4+^ and Co^2+^. However, our previous results on LCMO thin films prepared by sputtering, obtained from synchrotron XPS measurements, indicate that regardless of the structural strain (compressive or tensile) the oxidation states of Co and Mn ions are Co^2+^ and Mn^4+^ [32,33]. On the other hand, X-ray absorption spectroscopy analysis in LNMO samples prepared by PAD also confirm that the actual oxidation states are Ni^2+^ and Mn^4+^ [19]. In parallel with this, the obtained saturation magnetization values, slightly below the theoretical spin only saturation value, 6 μ_B_/f.u. in LCMO and close to 5 μ_B_/f.u. in LNMO, give further support to this idea, thus precluding the presence of several magnetic phases in the sample, in agreement with previous reports on samples prepared by sol-gel methods [34,35,36].

The origin of the specific shape of the *M*(*T*) curve is not clearly established and it should be related to the existence of some kind of magnetic disorder. This magnetic disorder may have two main different origins. On one side, the presence of anti-site disorder (ASD) (i.e., a portion of Co and Mn cations have their crystallographic sites interchanged) will trigger the appearance of Co^2+^-O-Co^2+^ and Mn^4+^-O-Mn^4+^ antiferromagnetic (AFM) interactions reducing the *Ms* value [19,28] and introducing competition of magnetic interactions, frustration and disorder. On the other side, multiple nucleation sites in the film can produce zones with alternating Co/Mn local ordering. When two of these zones merge together, at its interface deviations from the ideal Co/Mn ordering appear generating again Co^2+^-O-Co^2+^ and Mn^4+^-O-Mn^4+^ AFM interactions, giving rise to an antiphase boundary (APB) [37,38]. APBs appear as kind of domain walls between two FM domains, within which a Co^2+^/Mn^4+^ arrangement exists and their identification shows up as a sudden drop in the remnant magnetization at *H* = 0, as noticed in Figure 3b. Since *Ms* ≈ 5.7 μ_B_/f.u., i.e., nearby the ideal 6 μ_B_/f.u. spin-only saturation value (see Figure 3b), the amount of ASD in the sample should be very small, i.e., indicating that almost full B-B’ site cationic ordering has been achieved. On the other hand, Figure 3b also makes evident the existence of APBs (note the sudden drop of the remnant magnetization at *H* = 0). Therefore, it seems that the observed flattened *M*(*T*) curve should be associated with the existence of some amount of magnetic disorder in the sample mainly attributable in this case to APBs. Similar results were obtained in LCMO/STO samples prepared by conventional annealing [18]; however, *M*(*T*) curves in RTA seem to indicate a higher degree of magnetic disorder, which suggests a different microstructure, at least from the magnetic point of view. 

Post-growth annealing in an oxygen-rich atmosphere has proved to be an effective method to modify the microstructure reducing structural defects and oxygen vacancies in double perovskite ceramic samples and thin films [39,40]. The effect of a post-growth thermal treatment in a conventional tube furnace on the magnetic properties of the RTA grown sample (700 °C temperature, 4 h dwell time, 5 °C/min ramp, under an oxygen flow of 0.5 L/min) film LCMO/STO–1R, is evidenced in Figure 3c,d.

The temperature dependence of the magnetization, under an applied magnetic field of 1 kOe, shows that ferromagnetism decays at a slower rate, and that only a single *Tc* at a slightly higher temperature *T_c_* = 240 K with a sharper FM-PM transition is present. On the other hand, the sudden drop of the remnant magnetization at *H* = 0, indicative of the existence of APBs, has been completely suppressed while *Ms* ≈ 5.9 μ_B_/f.u., is slightly higher than in the as-grown sample. Therefore, the conventional post-growth annealing process is consistent with an overall suppression of the APBs reducing magnetic disorder as reflected in the *M*(*T*) curve.

**Figure 4:**
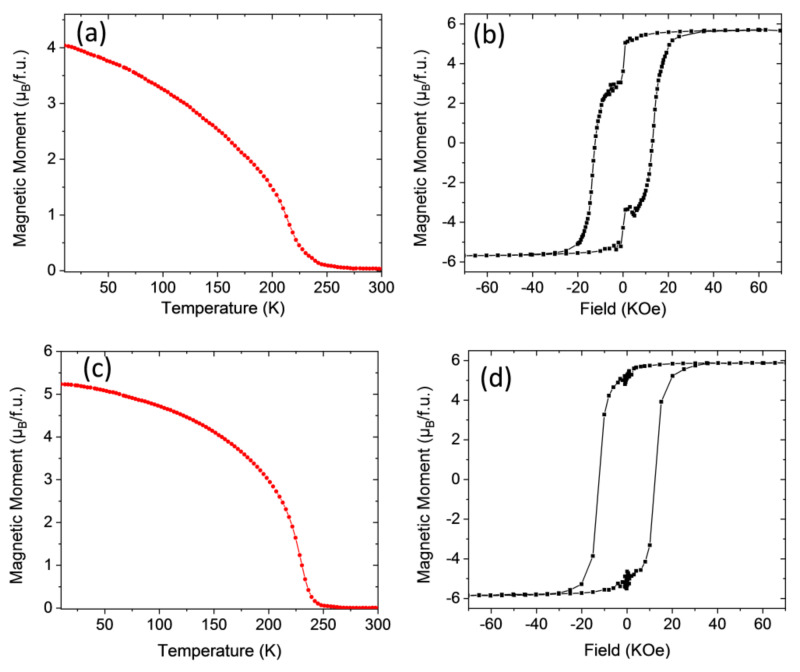
(**a**) Magnetization vs. temperature of the LCMO/LAO–1 film thermally treated in RTA conditions, measured after field cooling with a magnetic field of 1 kOe applied parallel to (100)-LAO, (in-plane). (**b**) M–H loop of the same sample recorded at 10 K by applying the magnetic field H in-plane. (**c**) Temperature dependence of the magnetization under an in-plane applied magnetic field of 1 kOe, of sample LCMO/LAO–1R. (**d**) M–H loop of sample LCMO/LAO–1R recorded at 10 K by applying the magnetic field H in-plane.

Similar results are obtained in the case of LCMO samples grown on top of LAO substrates as can be appreciated in Figure 4. Figure 4a shows the field cooled temperature dependence of the magnetization *M*(*T*) under an applied external magnetic field of 1 kOe in the in-plane (IP) configuration for the LCMO/LAO–1 sample processed in a static oxygen atmosphere with a heating ramp of 20 °C/s and a dwell time of 10 min at 900 °C. Compared to LCMO/STO samples, the *M*(*T*) curve is featureless but flattened with *Tc* ≈ 225 K, indicative of a smaller amount of magnetic disorder (see Figure 3a). The magnetic hysteresis loop recorded at 10 K, and displayed in Figure 4b, shows a magnetization saturation value near 5.7 μ_B_/f.u., slightly smaller than in a fully ordered sample and indicating almost full cationic ordering. Moreover, the typical drop of the remnant magnetization at *H* = 0, signature of APBs, is also observed, thus indicating that magnetic disorder is mainly introduced by APBs as in the previous case. 

A post-growth thermal treatment in oxygen flow, sample LCMO/LAO–1R (700 °C temperature, 4 h dwell time, 5 °C/min ramp, and oxygen flow of 0.5 L/min) equivalent to that performed in LCMO/STO samples, clearly modifies the *M*(*T*) curve, showing a slower decay rate of the magnetization and an increase of *Tc* of up to about 240 K (see Figure 4c). At the same time, the magnetic hysteresis loop recorded at 10 K and displayed in Figure 4d makes it evident that the annealing has fully suppressed APBs, while a slight increase of the saturation magnetization *Ms* value to about 5.8 μ_B_/f.u. was also found.

**Figure 5:**
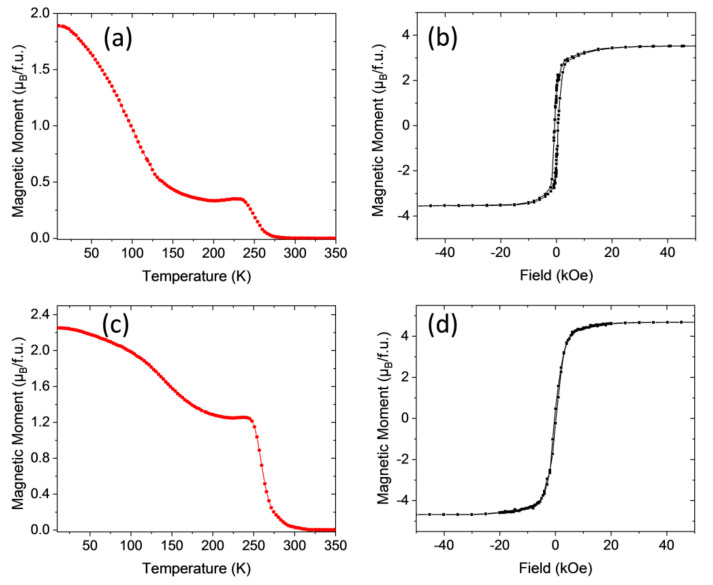
(**a**) Magnetization vs. temperature of an LNMO/STO−1 film thermally treated in RTA conditions, measured after field cooling with a magnetic field of 1 kOe applied parallel to (100)-SrTiO_3_, (in-plane). (**b**) M–H loop of the same sample recorded at 10 K by applying the magnetic field H in-plane. (**c**) Temperature dependence of the magnetization under an in-plane applied magnetic field of 1 kOe, of sample LNMO/STO−1R. (**d**) M–H loop of sample LNMO/STO−1R recorded at 10 K by applying the magnetic field H in-plane.

We move now to the analysis of the LNMO system. As evidenced in our previous studies in LNMO samples prepared by using conventional annealing processes [19], full B-B’ site cationic ordering is not achieved basically because the ionic radii difference between Ni^2+^ and Mn^4+^ is too small (16.0 pm as compared to 21.5 pm for Co^2+^ and Mn^4+^ [41]), so the amount of ASDs is substantially larger than in the LCMO samples. Therefore, the magnetic disorder linked to the introduction of Ni^2+^-O^2−^-Ni^2+^ and Mn^4+^-O^2−^-Mn^4+^ AFM interactions at anti-sites is larger than in LCMO samples, as can be clearly appreciated in Figure 5.

Figure 5a displays the temperature dependence of the magnetization *M*(*T*) under an applied external magnetic field of 1 kOe in the IP configuration for the LNMO/STO−1 thin film sample processed in static oxygen environment with a heating ramp of 20 °C/s and a dwell time of 20 min at 900 °C. As in the LCMO/STO case, the *M*(*T*) curve exhibits a non-monotonic behavior with a local minimum around *T* ≈ 200 K and a paramagnetic to ferromagnetic transition temperature *Tc* ≈ 255 K, which reflects the existence of magnetic disorder due to ASD involving Ni and Mn atoms [19,28]. The magnetic hysteresis loop recorded at 10 K is displayed in Figure 5b, and shows a depressed magnetization saturation value near 3.6–3.7 μ_B_/f.u., smaller than the value expected in a sample with full cationic ordering (5 μ_B_/f.u.), thus corroborating the existence of ASDs. It is worth mentioning here that the existence of several ASDs in the sample precludes the formation of APBs, since the magnetic disorder already generated blurs out the clear frontiers between zones with inverted Ni/Mn ordering. 

As already observed in LCMO thin films, a post-growth thermal treatment in oxygen flow (800 °C temperature, 3 h dwell time, 5 °C/min ramp, and oxygen flow of 0.5 L/min), sample LNMO/STO−1R, clearly modified the *M*(*T*) curve, showing a slower decay rate of the magnetization and an increase of *Tc* up to about 270 K (see Figure 5c). At the same time, the magnetic hysteresis loop recorded at 10 K shown in Figure 5d displays an unequivocal increase in the saturation magnetization *Ms* value, up to about *Ms* ~ 4.3 μ_B_/f.u, closer to the value expected in a fully ordered sample, therefore indicating a reduction in the ASD disorder density induced by the post-growth annealing in an oxygen rich atmosphere. The reduction of the magnetic disorder introduced by ASDs can also be appreciated in the reduction of the coercive fields (see Figure 5b,d).

**Figure 6:**
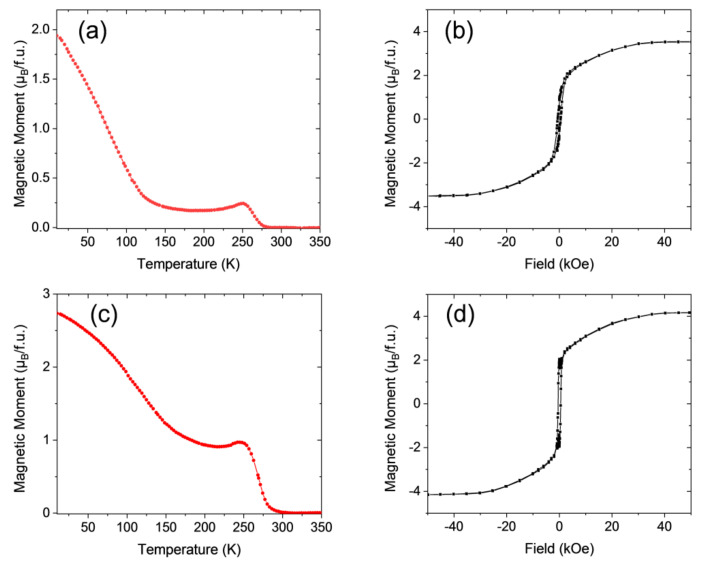
(**a**) Magnetization vs. temperature of a LNMO/LAO−1 epitaxial film thermally treated in RTA conditions, measured after field cooling with a magnetic field of 1 kOe applied parallel to (100)-LAO, (in-plane). (**b**) M–H loop of the same sample recorded at 10 K by applying the magnetic field H in-plane. (**c**) Temperature dependence of the magnetization under an in-plane applied magnetic field of 1 kOe, of sample LNMO/LAO−1R. (**d**) M–H loop of sample LNMO/LAO−1R recorded at 10 K by applying the magnetic field H in-plane.

The effects of the RTA process were also investigated in LNMO samples grown on LAO substrates and the results are compiled in Figure 6. The temperature dependence of the magnetization of sample LNMO/LAO−1 thermally treated with a heating ramp of 20 °C/s and a dwell time of 10 min at 900 °C in a static oxygen environment is shown in Figure 6a. As in the case of LNMO/STO samples, the *M*(*T*) curve exhibits a non-monotonic behavior with a pronounced local minimum at a temperature *T* ≈ 175 K and a paramagnetic to ferromagnetic transition temperature of *Tc* ≈ 265 K, indicative of the existence of a magnetic disorder. In agreement with this, the *M*(*H*) curve displayed in Figure 6b shows a slow approach to the saturation characteristic of a large magnetic disorder with a saturation value slightly below 3.5 μ_B_/f.u., which indicates the large amount of ASD that generates this disorder. This shape of *M*(*H*) curves was neither observed for LNMO/STO samples (see previous section), nor for LNMO/LAO samples grown in a conventional oven [19] and suggests a different microstructure. A post RTA thermal treatment under oxygen flow, sample LNMO/LAO−1R (800 °C temperature, 3 h dwell time, 5 °C/min ramp, and oxygen flow of 0.5 L/min), slightly improves the *M*(*T*) curve that is less flattened, and at the same time promotes an upward shift of the Curie temperature up to *Tc* = 275 K (see Figure 6c). In parallel, a slight increase of the *Ms* value up to about 4.1–4.2 μ_B_/f.u. was also observed, which would indicate a small decrease of the ASDs. However the line-shape of the *M*(*H*) curve is still clearly different from *M*(*H*) curves observed in the LCMO system and in that of LNMO/STO samples. 

**Figure 7:**
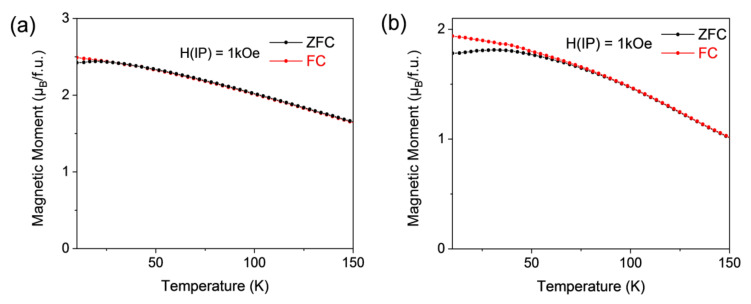
Zero-field-cooled (ZFC) and field-cooled (FC) magnetization curves measured at fields of 1 kOe for LNMO/STO epitaxial thin film samples grown by: (**a**) conventional thermal annealing (CTA) with 2 °C/min heating and cooling ramps, dwell time 30 min at 875 °C under oxygen flow, 0.4 L/min; (**b**) RTA conditions, 20 °C/s heating ramp, dwell time 20 min at 900 °C.

In the case of LNMO epitaxial films, the values of *Ms* obtained both in LNMO/STO and LNMO/LAO, around 4.3 μ_B_/f.u., after a post-RTA thermal treatment are among the best reported in the literature [35,36,40,42], corresponding to an ASD concentration below 10%. *M*(*T*) curves suggest a larger magnetic disorder. To gain a deeper insight into the nature of this disorder, *M*(*T*) curves have been analyzed after a zero field cooling-field cooling process (ZFC-FC). As shown in Figure 7 in RTA samples irreversibility at low T remains for applied magnetic fields well above the coercive field, while this irreversibility is fully suppressed in samples prepared by a conventional annealing process (see Supplementary Information, Figures S3 and S4). Therefore, LNMO samples prepared by RTA exhibit a spin glass-like behavior similar to that reported in [34]. 

As previously mentioned, both ASD and APB introduce Ni^2+^-O-Ni^2+^ and Mn^4+^-O-Mn^4+^ AFM interactions generating competition of magnetic interactions and magnetic disorder. Moreover, microstructural features (stacking faults, twins, vacancies) can contribute to pin magnetization making harder to achieve full magnetic saturation as observed in LNMO/LAO samples. 

Comparing the LNMO/STO and LNMO/LAO films, the results seem to indicate that cationic ordering is easier to attain in strained films, in good agreement with recently published results [43].

## 4. Conclusions

The influence of the annealing process on the microstructural and magnetic properties of LCMO and LNMO double perovskites prepared by PAD is analyzed. It is found that, irrespective of the structural strain, samples prepared by RTA exhibit similar values of *Tc* and *Ms* to their counterparts prepared by the conventional annealing process. Therefore, the heating ramp rate seems to have only a minor influence on the B-B’ site cationic ordering that is mainly controlled by charge difference and steric effects. In the case of the LCMO system, samples prepared by RTA present almost full B-B’ site cationic ordering with *Ms* values close to the spin-only theoretical value of 6 μ_B_/f.u. *M*(*H*) loops exhibit the sudden drop of the remnant magnetization at *H* = 0, indicative of the existence of APBs. In the case of the LNMO system, full B-B’ site cationic ordering is harder to attain since the ionic radii difference between Ni^2+^ and Mn^4+^ is too small. However, the values of *Ms* obtained both in LNMO/STO and LNMO/LAO, after a post-RTA thermal treatment, are among the best reported in the literature, suggesting state-of-the -art samples. The existence of an important amount of ASDs in LNMO samples precludes the appearance of APBs, since the magnetic disorder already generated blurs out the clear frontiers between zones with inverted Ni/Mn ordering. Nevertheless, it is worth mentioning that the magnetic behavior observed in samples prepared by RTA is different from that observed in samples prepared by a conventional annealing process, suggesting a different microstructure. Specifically, in the case of the LCMO system, it is observed that APBs in RTA samples are easily suppressed by a post-annealing treatment in contrast to what is observed in samples prepared by conventional annealing methods. However, X-ray microstructural analysis, i.e., rocking curves and reciprocal space maps, do not show relevant differences between samples prepared by RTA or by conventional processes. In samples prepared using a fast heating rate, a simultaneous nucleation occurs at high temperatures in a situation similar to that of isothermal annealing, which leads to a more homogeneous texture and grain size distribution and thus, there are few microstructural defects to pin APBs that are easily suppressed after a post-growth annealing. 

In the case of the LNMO system, as previously mentioned, the existence of ASDs precludes the formation of APBs, making it difficult to detect differences in the magnetic behavior between RTA and conventionally annealed samples. However, the approach to magnetic saturation in *M*(*H*) curves also suggest some differences. In fact, *M*(*T*) curves measured after a ZFC-FC process show the existence of irreversibility at low temperatures, for magnetic fields well above the coercive field, in RTA samples that is not detected in samples prepared by conventional annealing. This irreversibility indicates the existence of a magnetic disorder attributable to the disorder generated by the simultaneous nucleation of grains and their interaction with ASD in RTA samples. 

## Figures and Tables

**Table 1:** List of samples whose magnetic properties have been analyzed, with the corresponding RTA growth conditions and the parameters of the subsequent CTA treatment. The extracted values for *Tc* and *Ms* are also displayed.

Sample	Annealing Conditions Ramp, Temperature, Dwell Time, Oxygen	Curie Temperature, *Tc* (K)	Saturation Magnetization, *Ms* (μ_B_/f.u.)
LCMO/STO–1	RTA 20 °C/s, 900 °C, 20 min, static oxygen	230	5.7
LCMO/STO–1R	CTA 5 °C/min, 700 °C, 240 min, 0.5 L/min O_2_	240	5.9
LCMO/LAO–1	RTA 20 °C/s, 900 °C, 10 min, static oxygen	225	5.7
LCMO/LAO–1R	CTA 5 °C/min, 700 °C, 240 min, 0.5 L/min O_2_	240	5.8
LNMO/STO−1	RTA 20 °C/s, 900 °C, 20 min, static oxygen	255	3.7
LNMO/STO−1R	CTA 5 °C/min, 800 °C, 180 min, 0.5 L/min O_2_	270	4.3
LNMO/LAO−1	RTA 20 °C/s, 900 °C, 10 min, static oxygen	265	3.5
LNMO/LAO−1R	CTA 5 °C/min, 800 °C, 180 min, 0.5 L/min O_2_	275	4.2

## Supplementary Materials

The following are available online at https://www.mdpi.com/1996-1944/13/21/4966/s1, Figure S1: High-resolution θ/2θ x-ray diffraction (XRD) scans of the (0 0 2) reflections comparing thin films grown by conventional thermal annealing (CTA) and rapid thermal annealing (RTA). Figure S2: Rocking curves of the (0·0·2) reflections of La_2_NiMnO_6_/SrTiO3 thin film, and (b) La_2_NiMnO_6_/LaAlO_3_ thin film. Figure S3: Zero-field-cooled (ZFC) and field-cooled (FC) magnetization curves measured at fields of 100 Oe and 1 kOe for two La_2_NiMnO_6_ epitaxial thin film samples, grown by rapid thermal annealing (RTA). Figure S4: Zero-field-cooled (ZFC) and field-cooled (FC) magnetization curves measured at fields of 100 Oe and 1 kOe for two La_2_NiMnO_6_ epitaxial thin film samples, grown by conventional thermal annealing (CTA).
