# Supplementary material for: Rapid Thermal Annealing of Double Perovskite Thin Films Formed by Polymer Assisted Deposition
Source: Materials (Basel). 2020 Nov 4;13(21):4966. doi: 10.3390/ma13214966 (PMC7662915; doi:10.3390/ma13214966)
Supplement: Supplementary file 1 [file materials-13-04966-s001.pdf]

Supplementary Material

Rapid Thermal Annealing of Double Perovskite Thin Films Formed by Polymer Assisted Deposition

Hailin Wang, Carlos Frontera, Benjamín Martínez* and Narcís Mestres*

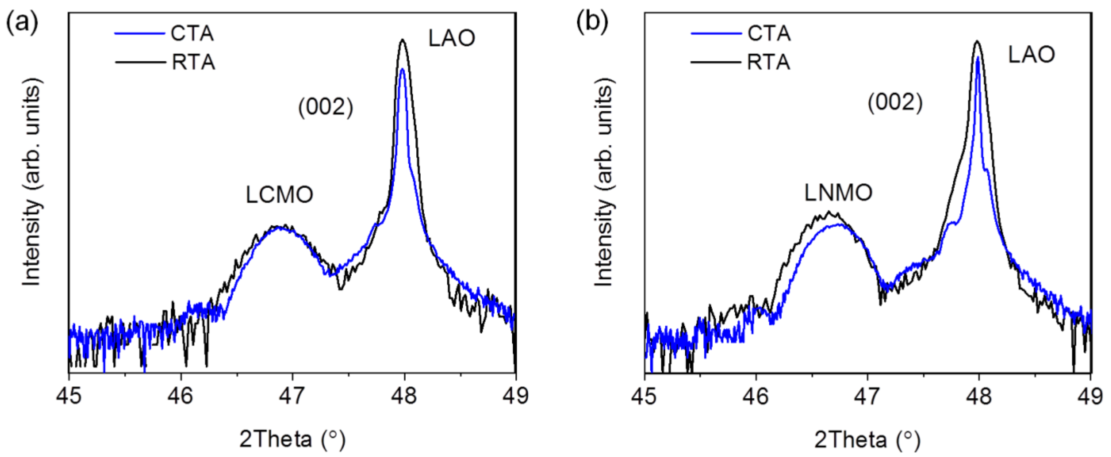

Figure S1. High-resolution θ/2θ x-ray diffraction (XRD) scans of the (0 0 2) reflections comparing thin films grown by conventional thermal annealing (CTA) and rapid thermal annealing (RTA). (**a**) LCMO/LAO and (**b**) LNMO/LAO thin films. The slightly different strained state observed between the RTA and CTA LNMO/LAO thin films may be due to small differences on thickness or strain relaxation of the films.

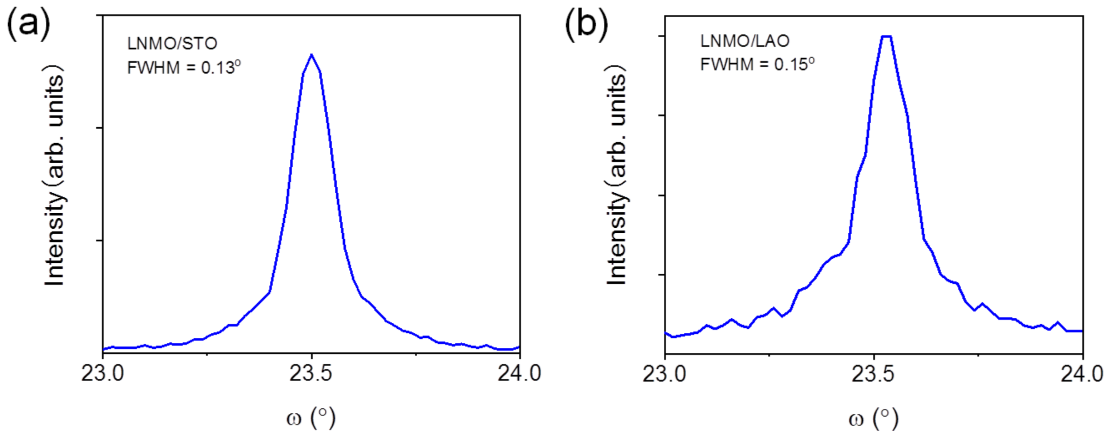

Figure S2. Rocking curves of the (0·0·2) reflections of (**a**) LNMO/STO thin film, and (**b**) LNMO/LAO thin film. The full width at half maximum (FWHM) values indicates the good crystallinity of the samples with some mosaicity.

Zero field cooled-field cooled (ZFC-FC) magnetization curves for different La_2NiMnO_6 (LNMO) epitaxial films prepared by rapid thermal annealing (RTA), measured at 100 Oe and 1 kOe are shown in Figure S3. For low fields (below coercive field *Hc*) irreversibility between ZFC and FC magnetization branches extends down to low temperatures. However, on increasing the applied field to 1 kOe, i.e. a field larger than *Hc*, the irreversibility is fully suppressed in samples prepared by conventional annealing process (see Figure S4), while it persists below about 40 K in the case of samples prepared by RTA (see Figure S3a).

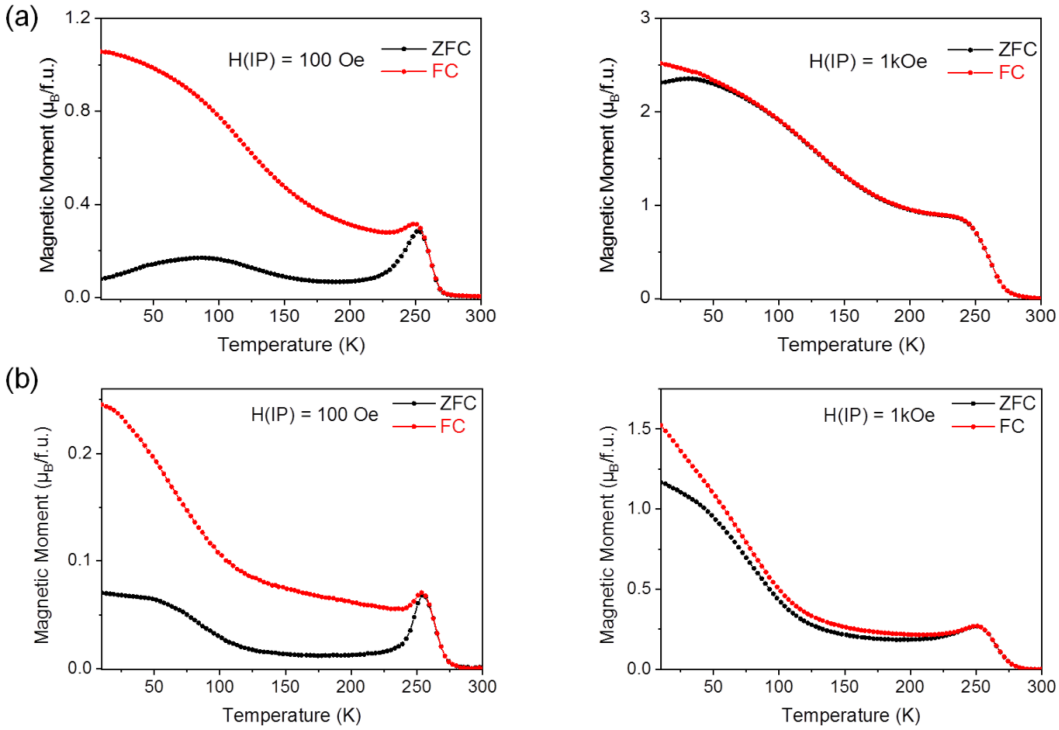

Figure S3. Zero-field-cooled (ZFC) and field-cooled (FC) magnetization curves measured at fields of 100 Oe and 1 kOe for LNMO epitaxial thin film samples grown by Rapid Thermal Annealing (RTA). (**a**) LNMO/STO film thermally treated in RTA conditions (20 °C/s heating ramp, dwell time 20 min at 900 °C); (**b**) LNMO/LAO epitaxial film thermally treated in RTA conditions (20 °C/s heating ramp, dwell time 10 min at 900 °C).

The existence of this low temperature irreversibility, for fields above *Hc*, in RTA samples is indicative of the existence of a higher magnetic disorder. This magnetic disorder should be attributable to the disorder generated by the simultaneous nucleation of grains in RTA samples and their interaction with anti site disorder (ASD). ASDs promote the appearance of Ni^{2+}-O-Ni^{2+} and Mn^{4+}-O-Mn^{4+} antiferromagnetic (AFM) interactions mimicking a spin glass-like behavior. Variations in the irreversibility between the ZFC and FC magnetization branches at low temperature should be somehow correlated with the amount of ASDs in the structure. In the case of LNMO/LAO with a higher degree of disorder irreversibility between ZFC and FC branches extends to higher temperatures (see Fig. S3b) even the global behavior is the same.

As pointed out by Choudhury *et al*. [1], the random crystallographic occupation sets the stage for mixed ferromagnetic (FM) and AFM interactions between the transition metal cations and in turn, leads to inhomogeneous magnetic behavior in La_2NiMnO_6. In a perfectly ordered double perovskite, the magnetic exchange is governed by the FM Ni^{2+}-O-Mn^{4+} interaction. With the occurrence of site disorder, additional AFM Ni^{2+}-O-Ni^{2+} and Mn^{4+}-O-Mn^{4+} paths are introduced. The spin glass like behavior that appears at low temperatures has its origin in the multiple exchange paths that arise due to mixed interactions [1, 2]. The competition between the FM and AFM interactions is the origin of the magnetic frustration which results in a spin glass-like state.

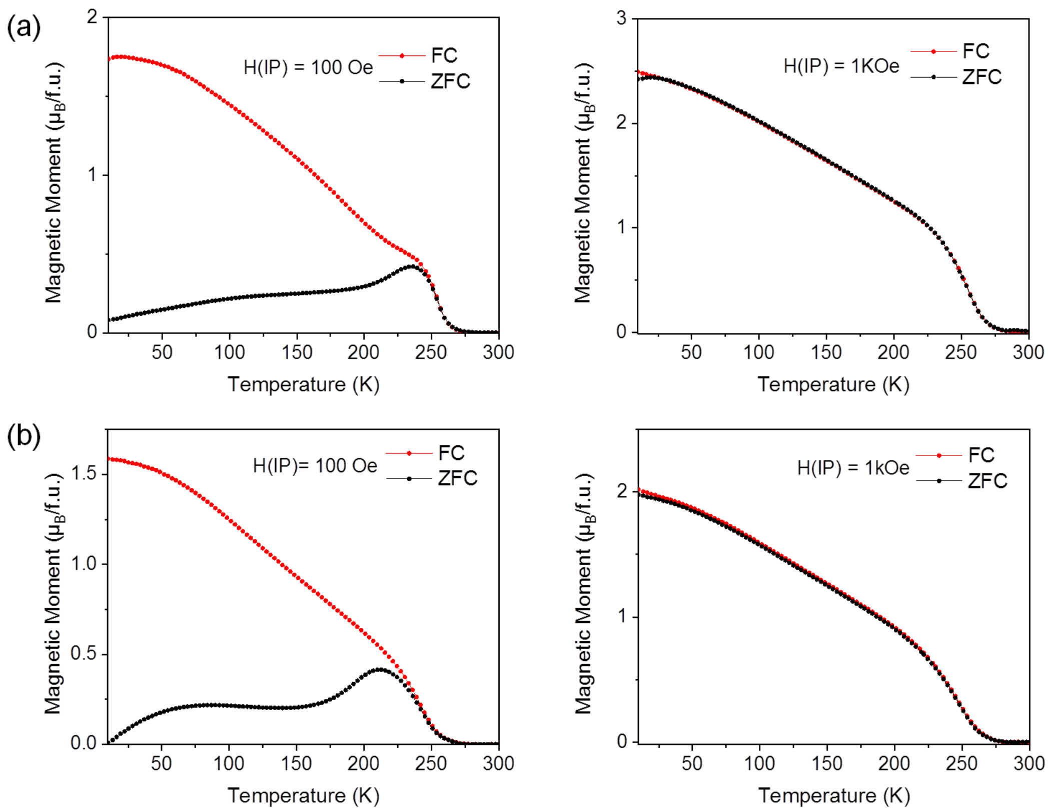

Figure S4. Zero-field-cooled (ZFC) and field-cooled (FC) magnetization curves measured at fields of 100 Oe and 1 kOe for LNMO epitaxial thin film samples grown by conventional thermal annealing (CTA). (**a**) LNMO/STO film thermally treated with 2 °C/min heating and cooling ramps, dwell time 30 min at 875°C under oxygen flow, 0.4 l/min; (**b**) LNMO/LAO epitaxial film thermally treated at the same conditions as previous sample.

References

1. Choudhury, D.; Mandal, P.; Mathieu, R.; Hazarika, A.; Rajan, S.; Sundaresan, A.; Waghmare, U. V.; Knut, R.; Karis, O.; Nordblad, P.; Sarma, D. D., Near-room-temperature colossal magnetodielectricity and multiglass properties in partially disordered La_2NiMnO_6. *Phys. Rev. Lett.* **2012,** 108, 127201. doi: 10.1103/PhysRevLett.108.127201.
2. Devi Chandrasekhar, K.; Das, A.K.; Venimadhav, A., Spin glass behavior and extrinsic origin of magnetodielectric effect in non-multiferroic La_2NiMnO_6 nanoparticles. *J. Phys.: Condens. Matter* **2012**, 24, 376003. doi:10.1088/0953-8984/24/37/376003.
